# Effective Biophysical Modeling of Cell Free Transcription and Translation Processes

**DOI:** 10.3389/fbioe.2020.539081

**Published:** 2020-11-26

**Authors:** Abhinav Adhikari, Michael Vilkhovoy, Sandra Vadhin, Ha Eun Lim, Jeffrey D. Varner

**Affiliations:** Robert Frederick Smith School of Chemical and Biomolecular Engineering, College of Engineering, Cornell University, Ithaca, NY, United States

**Keywords:** systems biology, synthetic biological circuits, cell free, mathematical modeling, simulation

## Abstract

Transcription and translation are at the heart of metabolism and signal transduction. In this study, we developed an effective biophysical modeling approach to simulate transcription and translation processes. The model, composed of coupled ordinary differential equations, was tested by comparing simulations of two cell free synthetic circuits with experimental measurements generated in this study. First, we considered a simple circuit in which sigma factor 70 induced the expression of green fluorescent protein. This relatively simple case was then followed by a more complex negative feedback circuit in which two control genes were coupled to the expression of a third reporter gene, green fluorescent protein. Many of the model parameters were estimated from previous biophysical studies in the literature, while the remaining unknown model parameters for each circuit were estimated by minimizing the difference between model simulations and messenger RNA (mRNA) and protein measurements generated in this study. In particular, either parameter estimates from published studies were used directly, or characteristic values found in the literature were used to establish feasible ranges for the parameter estimation problem. In order to perform a detailed analysis of the influence of individual model parameters on the expression dynamics of each circuit, global sensitivity analysis was used. Taken together, the effective biophysical modeling approach captured the expression dynamics, including the transcription dynamics, for the two synthetic cell free circuits. While, we considered only two circuits here, this approach could potentially be extended to simulate other genetic circuits in both cell free and whole cell biomolecular applications as the equations governing the regulatory control functions are modular and easily modifiable. The model code, parameters, and analysis scripts are available for download under an MIT software license from the Varnerlab GitHub repository.

## 1. Introduction

Cell free systems are a widely used research tool in systems and synthetic biology and a promising platform for the manufacturing of proteins and chemicals (Vilkhovoy et al., 2020). A distinctive feature of cell free systems is the absence of cellular growth and maintenance, thereby allowing the direct allocation of carbon and energy resources toward a product of interest. Cell free systems are also more amenable than living systems to observation and manipulation, hence allowing rapid tuning of reaction conditions. Arguably, the most widely used cell free technology is cell free protein synthesis (CFPS), an *in vitro* platform for protein transcription (TX) and translation (TL). Transcription and translation, the processes by which information stored in DNA is converted to a working protein, are at the center of metabolism and signal transduction programs important to biotechnology and human health. For example, evolutionarily conserved developmental programs such as the epithelial to mesenchymal transition (EMT) (Thiery, 2003), or retinoic acid induced differentiation (Nilsson, 1984), rely on multiple rounds of highly coordinated gene expression. From the perspective of biotechnology, even relatively simple industrially important organisms such as *Escherichia coli*, have intricate transcriptional regulatory networks which control the metabolic state of the cell in response to changing nutrient conditions (Orth et al., 2010; Vilkhovoy et al., 2016). Understanding the dynamics of regulatory networks can be greatly facilitated by mathematical models. A majority of these models fall into three categories: logical, continuous, and stochastic models (Karlebach and Shamir, 2008). Logical models such as Boolean networks (Glass and Kauffman, 1973) developed using a variety of approaches and data (Pratapa et al., 2020) represent the state of each network entity as a discrete variable, provide a quick but qualitative description of the behavior of the regulatory network. Linear and non-linear ordinary differential equation (ODE) models fall into the second category, and they generally provide a detailed picture of the network dynamics, although they can be non-physical models, e.g., relying on a gene signal perspective (Bonneau et al., 2006). Lastly, stochastic models describe the interactions between individual molecules, and discrete reaction events (McAdams and Arkin, 1997; Mettetal et al., 2006; Kaufmann and van Oudenaarden, 2007; Raj and van Oudenaarden, 2008). Model choice depends on criteria such as speed, the level of detail required and the quantity of experimental data available to estimate the model parameters. While the end goal of the models might be to accurately predict *in vivo* behavior, living systems do not necessarily provide an ideal experimental platform. For example, although there have been significant advancements in metabolomics (e.g., Park et al., 2016), the rigorous quantification of intracellular messenger RNA (mRNA) copy number or protein abundance remains challenging. Toward this challenge, cell free systems offer several advantages for the study of transcription and translation processes.

Cell free biology has historically been an important tool to study the fundamental biological mechanisms involved with gene expression. In the 1950s, cell free systems were used to explore the incorporation of amino acids into proteins (Borsook, 1950; Winnick, 1950a,b), and the role of adenosine triphosphate (ATP) in protein production (Hoagland et al., 1956). Further, *E. coli* extracts were used by Nirenberg and Matthaei in 1961 to demonstrate templated translation (Matthaei and Nirenberg, 1961; Nirenberg and Matthaei, 1961), leading to a Nobel Prize in 1968 for deciphering the codon code. More recently, as advancements in extract preparation and energy regeneration have extended their durability, the usage of cell free systems has also expanded to both small- and large-scale biotechnology and biomanufacturing applications (Swartz, 2018; Silverman et al., 2019). Today, cell free systems have been implemented for therapeutic protein and vaccine production (Ng et al., 2012; Jaroentomeechai et al., 2018; Stark et al., 2019), biosensing (Soltani et al., 2018), genetic part prototyping (Moore et al., 2017) and minimal cell systems (Yue et al., 2019). The versatility of cell free systems offers a tremendous opportunity for the systems-level experimental and computational study of biological mechanism. For example, a number of ordinary differential equation based cell free models have been developed (Stögbauer et al., 2012; Mavelli et al., 2015; Matsuura et al., 2017; Doerr et al., 2019; Marshall and Noireaux, 2019). However, despite the obvious advantages offered by a cell free system, experimental determination of the kinetic parameters for these models is often challenging. For instance, the cell free modeling study of Horvath and coworkers (which included a description of transcription and translation, and the underlying metabolism supplying energy and precursors for transcription and translation), had over 800 unknown model parameters (Horvath et al., 2020). Moreover, the construction, identification and validation of the Horvath model took well over a year to complete. Thus, constructing, identifying and validating biophysically motivated cell free models, which are simultaneously manageable, is a key challenge. Toward this challenge, effective modeling approaches which coarse grain biological details but remain firmly rooted in a biophysical perspective, could be an important tool.

In this study, we developed an effective biophysical modeling approach to simulate cell free transcription and translation processes. The model used classical biophysical arguments to formulate kinetic expressions for the rates of transcription and translation. These rates were then used in material balance equations (ordinary differential equations) to simulate the mRNA and protein concentration as a function of time for different cell free genetic circuits. The model was effective as it neglected potentially important mechanistic factors, and the integration of transcription and translation with metabolism. For example, the model did not consider how the transcription and translation rate was influenced by the availability of metabolic resources, e.g., energy or building block concentration. Nor did the model consider potentially important biology, for example the role of elongation factors or protein folding chaperones (among many other potentially important factors). We tested this approach by comparing simulations of two cell free synthetic circuits with messenger RNA (mRNA) and protein measurements (deGFP) generated in this study using the *E. coli* based myTXTL cell free system. First, we considered a simple circuit (C1) in which endogenous sigma factor 70 (σ_70_) induced the expression of a fast maturing dual emission green fluorescent protein variant (deGFP). This relatively simple case was then followed by a more complex negative feedback circuit (C2) where two control genes were coupled to the expression of deGFP. The second circuit is an extension of the first, with the presence of additional regulatory elements. Characteristic values for many of the model parameters were estimated from published biophysical studies or took the form of corrections to order of magnitude literature estimates, while the remaining unknown model parameters for each circuit were estimated by minimizing the difference between simulated and measured mRNA and protein concentrations. In particular, either parameter estimates from published studies were used directly, or characteristic values found in the literature were used to establish feasible ranges for the parameter estimation problem. Next, in order to provide a detailed insight into the influence of individual model parameters on the expression dynamics of each circuit, Morris sensitivity analysis was employed. For C1, the sensitivity results were informative, but expected. However, for C2, the analysis hierarchically stratified the parameters (and associated model species) into local vs. global categories. For example, parameters that controlled the abundance of lambda phage repressor protein (cI-ssrA), a master circuit regulator in C2, were globally important as they influenced all other species. On the other hand, the parameters that influenced deGFP levels (the endpoint of both circuits) were only locally important to deGFP. Taken together, the effective biophysical modeling approach captured the expression dynamics, including the transcription dynamics, for two synthetic cell free circuits. While, we considered only two circuits here, this approach could potentially be extended to simulate other genetic circuits in both cell free and whole cell biomolecular applications. The model code, parameters, and analysis scripts are available under an MIT software license from the Varnerlab GitHub repository^1^.

## 2. Materials and Methods

### 2.1. Cell Free Protein Synthesis Reactions

The cell free protein synthesis (CFPS) reactions were carried out using the myTXTL Sigma 70 Master Mix (Arbor Biosciences) in 1.5 mL Eppendorf tubes. The working volume of all the reactions was 12 μL, composed of the Sigma 70 Master Mix (9 μL) and the plasmids (3 μL total): P70a-deGFP (5 nM) for the single-gene system; P70a-deGFP-ssrA (8 nM), P70a-S28 (1.5 nM), and P28a-cI-ssrA (1 nM) for the negative feedback circuit. The plasmids were bought in lyophilized form (Arbor Biosciences) and purified using QIAprep Spin Miniprep Kit (Qiagen) using cell lines DH5-Alpha (for P28a-cI-ssrA) or KL740 (for P70a-deGFP, P70a-deGFP-ssrA, and P70a-S28). The CFPS reactions were incubated at 29°C.

### 2.2. mRNA and Protein Quantification

Following each CFPS run, the total RNA was extracted from 1 μL of the reaction mixture using PureLink RNA Mini Kit (Thermo Fisher Scientific) and stored at −80°C. The quantitative RT-PCR reactions were done using Applied Biosystems™ TaqMan™ RNA-to-CT™ 1-Step Kit and Custom TaqMan Gene Expression Assays (Thermo Fisher Scientific). An mRNA standard curve was used to determine absolute mRNA concentrations for each of the samples. The mRNA standards were prepared as follows: separate CFPS reactions for 5 nM of plasmids (P70a-S28, P70a-deGFP, and P70a-deGFP-ssrA) were carried out for 2 h. Total RNA was extracted using the full reaction volume. This was followed by the removal of 16S and 23S rRNA using the MICROBExpress™Bacterial mRNA Enrichment Kit (Life Technologies Corporation). Lastly, the MEGAclear™Kit (Life Technologies Corporation) was used to further purify the mRNA. The mRNA concentrations were determined using the Qubit™RNA assay kit (ThermoFisher Scientific). At least three technical replicates were performed for each standard. The concentration of cI-ssrA mRNA was quantified using the deGFP-ssrA mRNA standard. Green fluorescent protein (deGFP) fluorescence was measured using the Varioskan Lux plate reader at 488 nm (excitation) and 535 nm (emission). At the end of the CFPS run, 3 μL of the reaction mixture was diluted in 33 μL phosphate buffered saline (PBS) and stored at −80°C. The fluorescence was measured in triplicate with 10 μL each of this mixture. For all measurements, at least three biological replicates were performed.

### 2.3. Synthetic Circuit Architecture

**Figure 1:**
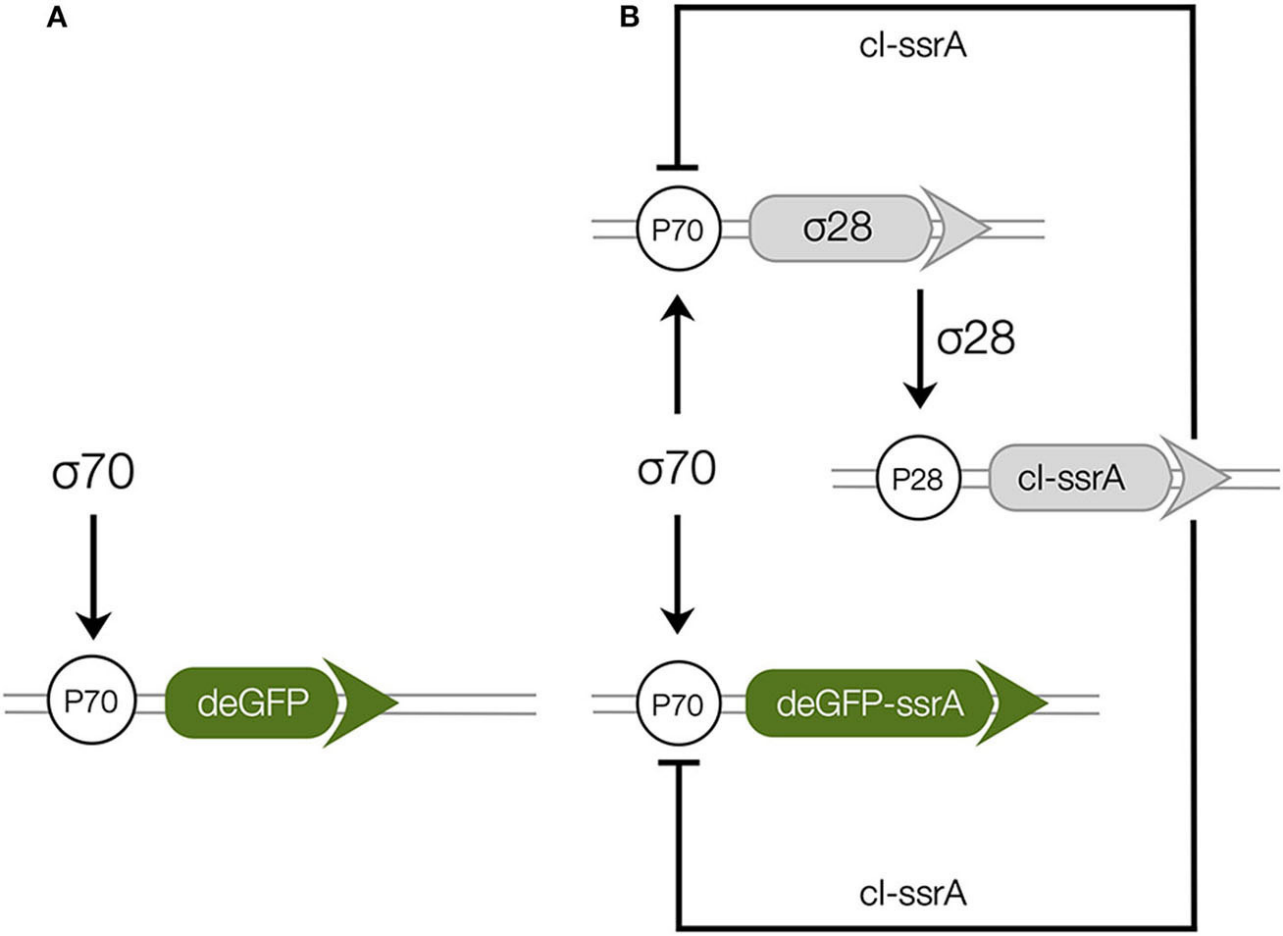
Schematic of the cell free gene expression circuits used in this study. **(A)**: Sigma factor 70 (σ_70_) induced expression of deGFP. **(B)**: The circuit components encode for a negative feedback loop motif. Sigma factor 28 and deGFP-ssrA genes on the P70a promoters are expressed first because of the endogenous presence of sigma 70 factor in the extract. Sigma factor 28, once expressed, induces the P28a promoter, turning on the expression of the cI-ssrA gene which represses the P70a promoter. The circuit is modified from a previous study (Garamella et al., 2016) by including an ssrA degradation tag on the cI gene.

The two genetic circuits (C1 and C2) used in this study were based upon the bacterial sigma factor regulatory system (Figure 1). Sigma factor 70 (σ_70_), endogenously present in the extract, was the primary driver of each circuit. In C1, σ_70_ induced green fluorescent protein (deGFP) expression was explored in the absence of additional regulators or protein degradation (Figure 1A). In C2, σ_70_ induced the expression of sigma factor 28 (σ_28_) and deGFP-ssrA (Figure 1B). Sigma 28 induced the expression of the lambda phage repressor protein cI-ssrA, which was under the σ_28_ responsive P28 promoter. The cI-ssrA protein repressed the P70a promoter, thereby down-regulating σ_28_ and deGFP-ssrA transcription (Marshall and Noireaux, 2018). Simultaneously, the C-terminal ssrA degradation tags present on the deGFP and cI proteins were recognized by the endogenous ClpXP protease system in the cell free extract, thereby promoting the degradation of these proteins into peptide fragments (Flynn et al., 2003; Garamella et al., 2016). In addition, messenger RNAs (mRNAs) were always subject to degradation due to the presence of degradation enzymes in the extract (Karzbrun et al., 2011; Garamella et al., 2016). Taken together, the interactions of the components manifested in an accumulation of deGFP protein for C1, and a pulse signal of deGFP-ssrA in C2. Studying C1 allowed us to estimate parameters governing the interaction of σ_70_ with the P70a promoter. Whereas, the C2 allowed us to characterize the interaction of σ_28_ with the P28 promoter, the strength of the transcriptional repression by cI-ssrA, and the kinetics of protein degradation by the endogenous ClpXP protease system. Finally, both circuits tested the effective model formulation for the transcription and translation rates.

### 2.4. Formulation and Solution of Model Equations

Consider a cell free synthetic circuit composed of the genes G=1,2,…,N. Each gene in the circuit is described by two differential equations, one for mRNA (*m*_*j*_) and a second for the corresponding protein (*p*_*j*_):

(1) $$\begin{array}{l} {ṁ}_{j}=r_{X,j}u_{j}\left(\ldots \right)-\theta_{m,j}m_{j}\;j=1,2,\ldots ,N \end{array}$$

(2) $$\begin{array}{l} {ṗ}_{j}=r_{L,j}w_{j}\left(\ldots \right)-\theta_{p,j}p_{j} \end{array}$$

The term *r*_*X,j*_*u*_*j*_(…) in the mRNA balance, which denotes the regulated rate of transcription for gene j, is the product of a kinetic limit *r*_*X,j*_ (nM h^−1^) and a transcription control function 0 ≤ *u*_*j*_(…) ≤ 1 (dimensionless). Similarly, the rate of translation of mRNA j, denoted by *r*_*L,j*_*w*_*j*_, is also the product of the kinetic limit of translation (nM h^−1^) and a translational control term 0 ≤ *w*_*j*_(…) ≤ 1 (dimensionless). Lastly, θ_⋆, *j*_ denotes the first-order rate constant (h^−1^) governing degradation of protein and mRNA. The model equations, encoded in the Julia programming language (Bezanson et al., 2017), were automatically generated using the JuGRN tool^2^. The model equations were solved numerically using the Rosenbrock23 routine of the DifferentialEquations.jl package (Rackauckas and Nie, 2017).

#### 2.4.1. Transcription and Translation Kinetic Limits

The key idea behind the transcription and translation kinetic limit expressions is that the polymerase (or ribosome) acts as a pseudo-enzyme; it binds a gene (or message), reads the gene (or message), and then dissociates. Thus, we used a strategy similar to classical enzyme kinetics to derive expressions for *r*_*X,j*_ (or *r*_*L,j*_); we proposed a set of elementary reactions for transcription and translation, one of which we assumed was rate limiting, and then invoked the pseudo state assumption for each intermediate complex to develop the overall rate expression. Following this approach, the details of the derivation of *r*_*X,j*_ (or *r*_*L,j*_) are given in the Supplementary Materials. The transcription kinetic limit *r*_*X,j*_ is given by:

(3) $$\begin{array}{l} r_{X,j}=V_{X,j}^{max}\left(\frac{G_{j}}{\tau_{X,j}K_{X,j}+\left(1+\tau_{X,j}\right)G_{j}+O_{X,j}}\right) \end{array}$$

where $V_{X,j}^{max}$ denotes the maximum transcription rate (nM/h) of gene *j*, $G_{j}$ denotes the concentration of gene *j* (nmol/L), *K*_*X,j*_ denotes the saturation constant for transcription of gene *j* (nmol/L), τ_*X,j*_ denotes the time constant for transcription (dimensionless) and:

(4) $$\begin{array}{l} O_{X,j}=\overset{N}{\underset{i=1,j}{\sum }}\frac{K_{X,j}\tau_{X,j}}{K_{X,i}\tau_{X,i}}\left(1+\tau_{X,i}\right)G_{i} \end{array}$$

denotes the coupling of the transcription of gene *j* with the other genes in the system through competition for RNA polymerase.

In a similar way, we developed an expression for the translational kinetic limit:

(5) $$\begin{array}{l} r_{L,j}=V_{L,j}^{max}\left(\frac{m_{j}}{\tau_{L,j}K_{L,j}+\left(1+\tau_{L,j}\right)m_{j}+O_{L,j}}\right) \end{array}$$

where $V_{L,j}^{max}$ denotes the maximum translation rate (nM/h), *K*_*L,j*_ denotes the saturation constant for translation of mRNA message *j* (nmol/L), τ_*L,j*_ denotes the time constant for translation of message *j* (dimensionless) and:

(6) $$\begin{array}{l} O_{L,j}=\overset{N}{\underset{i=1,j}{\sum }}\frac{K_{L,j}\tau_{L,j}}{K_{L,i}\tau_{L,i}}\left(1+\tau_{L,i}\right)m_{i} \end{array}$$

describes the coupling of the translation of mRNA *j* with other messages in the system because of kinetic competition for available ribosomes. The saturation and time constants for each case (which are unknown and must be estimated from experimental data) are defined in the Supplementary Materials. Lastly, in this study, the $O_{X,j}$ and $O_{L,j}$ terms were neglected as both circuits had either only one, or a small number of genes.

The maximum transcription rate $V_{X,j}^{max}$ was formulated as:

(7) $$\begin{array}{l} V_{X,j}^{max}\equiv R_{X,T}\left(\frac{{\dot{v}}_{X}}{l_{G,j}}\right) \end{array}$$

where *R*_*X,T*_ denotes the total RNA polymerase concentration (nM), ${\dot{v}}_{X}$ denotes the RNA polymerase elongation rate (nt/h) and *l*_*G, j*_ denotes the length of gene *j* in nucleotides (nt). Similarly, the maximum translation rate $V_{L,j}^{max}$ was formulated as:

(8) $$\begin{array}{l} V_{L,j}^{max}\equiv K_{P}R_{L,T}\left(\frac{{\dot{v}}_{L}}{l_{P,j}}\right) \end{array}$$

where *R*_*L,T*_ denotes the total ribosome pool, *K*_*P*_ denotes the polysome amplification constant, ${\dot{v}}_{L}$ denotes the ribosome elongation rate (amino acids per hour), and *l*_*P, j*_ denotes the length of protein *j* (aa).

#### 2.4.2. Control Functions *u* and *w*

Values of the control functions *u*(…) and *w*(…) describe the regulation of transcription and translation. Ackers et al., borrowed from statistical mechanics to recast the *u*(…) function as the probability that a system exists in a configuration which leads to expression (Ackers et al., 1982). The idea of recasting *u*(…) as the probability of expression was also developed (apparently independently) by Bailey and coworkers in a series of papers modeling the *lac* operon (see Lee and Bailey, 1984). More recently, Moon and Voigt adapted a similar approach when modeling the expression of synthetic circuits in *E. coli* (Moon et al., 2012). The *u*(…) function is formulated as:

(9) $$\begin{array}{l} u{\left(\ldots \right)}_{j}=\frac{\underset{i\in \left\{\chi \right\}}{\sum }W_{i}f_{i}\left(\ldots \right)}{\underset{j\in C_{j}}{\sum }W_{j}f_{j}\left(\ldots \right)} \end{array}$$

where *W*_*i*_ (dimensionless) denotes the weight of configuration *i*, while *f*_*i*_(⋯) (dimensionless) is a binding function (taken to be a hill-type function) describing the fraction of bound activator/inhibitor for configuration *i*. The summation in the numerator of Equation (9) is over the set of promoter configurations leading to expression (denoted as χ), while the summation in the numerator is over the set of all possible configurations for gene *j* (denoted as $C_{j}$). Thus, *u*(…)_*j*_ can be thought of as the fraction of all possible configurations that lead to expression. The weights *W*_*i*_ are related to the Gibbs energy of configuration *i*: *W*_*i*_ = exp(−Δ*G*_*i*_/*RT*) where Δ*G*_*i*_ denotes the molar Gibbs energy for configuration *i* (kJ/mol), *R* denotes the ideal gas constant (kJ mol^−1^ K^−1^), and *T* denotes the system temperature (Kelvin) (Ackers et al., 1982). The value of the binding function depends on the concentrations of the different transcriptional elements and their dissociation constants. The temporal evolution of *u*, therefore, is tied to the dynamics of its transcriptional elements, and its value lies between 0 and 1. In the case of circuit C1, *u* did not vary during the course of the reaction because the concentration of its activator, σ_70_, was fixed. For this case, *u* approximately equalled 0.95. However, in the second circuit, C2, *u* varied with time because of the change in levels of σ_28_ and cI-ssrA proteins.

We accounted for the experimentally observed loss of translational activity through the translational control function *w*(…). Loss of translational activity could be a function of many factors, including depletion of metabolic resources. However, in this study, we modeled the loss of translational activity as an exponential decay process with half-life τ_*L*,1/2_:

(10) $$\begin{array}{l} \dot{\epsilon }=-\left(\frac{0.693}{\tau_{L,1/2}}\right)\epsilon \end{array}$$

where ϵ denotes the fraction of remaining translational activity. Initially we assumed translation to be fully active, ϵ(0) = 1. Solving equation (10) yields ϵ(*t*) = exp(−0.693·*t*/τ_*L*,1/2_). Over time, as the cell free reaction progressed, the translational activity decreased with a half-life τ_*L*,1/2_ which was estimated from experimental data. The translational control variable was then given by *w*_*i*_ = ϵ for all translation processes.

### 2.5. Estimation of Model Parameters

Model parameters were estimated from published studies, were specified by experimental conditions (Table 1) or were estimated by minimizing the squared difference between model simulations and messenger RNA (mRNA), or protein measurements generated in this study. For the P70-deGFP model (C1), 11 parameters were estimated, while 33 parameters were estimated for the negative feedback model (C2).

**Table 1:** Characteristic parameters for TX/TL model equations.

**Description**	**Parameter**	**Value**	**Units**	**Reference**
RNA polymerase concentration	*R*_*X,T*_	0.06–0.075	μM	*a*
Ribosome concentration	*R*_*L,T*_	<2.3	μM	*a,b*
σ_70_ concentration	σ_70_	<35	nM	*a*
initial σ_28_ concentration	σ_28_	<20	nM	*a*
Transcription elongation rate	${\dot{v}}_{X}$	12–30	nt/s	*a,d*
Translation elongation rate	${\dot{v}}_{L}$	1–2	aa/s	*a,b*
Transcription saturation coefficient	*K*_*X*_	0.036	μM	*i*
Polysome amplification constant	*K*_*P*_	10.0	constant	*e*
Transcription initiation time	$k_{I}^{X}$	22	s	*i*
Translation initiation time	$k_{I}^{L}$	1.5	s	*e*
Default mRNA degradation coefficient	θ_*m*_	3.75	h^-1^	*a*
Default protein degradation coefficient	θ_*p*_	0.462–1.89	h^-1^	*f,g*
Gene concentration σ_28_		1.5	nM	*c*
Gene concentration cI-ssrA		1.0	nM	*c*
Gene concentration deGFP-ssrA		8.0	nM	*c*
Gene length σ_28_		811	nt	*h*
Gene length cI-ssrA		850	nt	*h*
Gene length deGFP-ssrA		782	nt	*h*
Protein length σ_28_		240	aa	*h*
Protein length cI-ssrA		248	aa	*h*
Protein length deGFP-ssrA		237	aa	*h*

*Key to references used in the table: (a) Garamella et al. (2016), (b) Underwood et al. (2005), (c) set by experiment, (d) Kassavetis and Chamberlin (1981), (e) estimated in this study, (f) Niederholtmeyer et al. (2015) and Vilkhovoy et al. (2018), (g) Grilly et al. (2007), (h) calculated from plasmid sequence, (i) McClure (1980)*.

The minimization problem to estimate the unknown model parameters was structured as a multiobjective optimization problem in which each measured mRNA or protein trajectory was treated as a separate objective. The minimization problem was solved using the Pareto Optimal Ensemble Technique in the Julia programming language (JuPOETs) (Bassen et al., 2017). JuPOETs is a multiobjective optimization approach which integrates simulated annealing with Pareto optimality to estimate parameter values on or near the optimal tradeoff surface between *N* potentially competing objectives (squared difference between model simulations and experimental measurements). JuPOETs minimized a problem of the form:

(11) $$\begin{array}{l} \underset{k}{\min}E_{j}=\overset{T_{j}}{\underset{i=1}{\sum }}{\left({\hat{M}}_{ij}-x_{ij}\left(k\right)\right)}^{2}\;j=1,2,\ldots ,N \end{array}$$

subject to

(12) $$\begin{array}{l} ẋ=f\left(x,k\right) \end{array}$$

(13) $$\begin{array}{lll} L\leq & k & \leq U \end{array}$$

(14) $$\begin{array}{l} x\left(t_{o}\right)=x_{o} \end{array}$$

where Equation (12) denotes the model equations, Equation (13) denotes the parameter bounds, and Equation (14) denotes the initial conditions. The objective function(s) $E_{j}$ measured the squared difference between model simulations and experiment *j* (either a protein or mRNA trajectory). The symbol ${\hat{M}}_{ij}$ denotes an experimental observation at time index *i* from experiment *j*, while the symbol *x*_*ij*_ denotes the model simulation output at time index *i* from experiment *j*. The quantity *i* denotes the sampled time-index and $T_{j}$ denotes the number of time points for experiment *j*. For the P70-deGFP model (C1), $E_{1}$ corresponded to mRNA deGFP, while $E_{2}$ corresponded to the deGFP protein concentration. On the other hand, for the negative feedback model (C2), $E_{1}$ corresponded to mRNA deGFP-ssrA, $E_{2}$ to mRNA σ_28_, $E_{3}$ to mRNA cI-ssrA and $E_{4}$ to the deGFP-ssrA protein concentration. Lastly, we penalized accumulation of the cI-ssrA protein (unmeasured) reaching unrealistically high levels with a term of the form: $E_{5}$ = *C* × max(0, *x*_*cI*_ − *U*_*cI*_) where *C* denotes a penalty parameter (*C* = 1 × 10^5^), *x*_*cI*_ denotes the maximum simulated cI-ssrA protein concentration, and *U*_*cI*_ denotes a concentration upper bound (*U*_*cI*_ = 100μ*M*). This bound was chosen to be approximately five-fold higher than the protein levels observed in an uninhibited circuit (e.g., C1).

The lower and upper bounds for unknown model parameters were established from previously published studies, or from previous model analysis; parameter values estimated for the P70-deGFP model were also used to establish ranges for the negative feedback model. JuPOETs searched over Δ*G*_*i*_, *K*_*L*_, and τ_*L*,1/2_ values directly, while other unknown parameter values took the form of corrections to order of magnitude characteristic literature estimates. For example, we set the mRNA degradation rate constant (θ_*m*_) to a characteristic value taken from literature. Then, the degradation constant for any particular mRNA was represented as: θ_*m,i*_ = α_*i*_θ_*m*_, where α_*i*_ was an unknown (but bounded) modifier. In this way, we guaranteed the parameter search (and the resulting estimated parameters) were within a specified range of literature values. We used this procedure for all degradation constants (both mRNA and protein) and all time constants (for both transcription and translation). The baseline parameter values are given in Table 1. JuPOETs was run for 20 generations for both models, and all parameters sets with Pareto rank less than or equal to two were collected for each generation. The JuPOETs parameter estimation routine is encoded in the sa_poets_estimate.jl script in the model repositories.

JuPOETs uses a simulated annealing approach to generate candidate parameter solutions whose Pareto rank is then evaluated; ranks below a threshold are kept while higher rank solutions are discarded. Thus, all the advantages (and disadvantages) associated with simulated annealing have been inherited by JuPOETs; for example, the time required to generate a family of low rank solutions will be significantly longer than a derivative based approach. Beyond these specific performance issues, a unique pathology of JuPOETs is the use of Pareto rank as a surrogate for training error. JuPOETs attempts to find low rank solutions, but rank is a relative measure of the quality of a solution. Thus, during the early iterations, low rank solutions often have large errors. As the iteration count increases the approach tends to find low error solutions with low rank, however, for complex models the rate of convergence to these low rank low error solutions is slow. To address this concern, we periodically switch to single objective mode where we minimize the total training error (summation of all objective functions) instead of finding low rank solutions. The best solutions from single objective mode can then be used to restart the multi-objective calculation. This hybrid approach, which was used in this study, has previously been shown to increase the rate of finding low rank and low error solutions (see Bassen et al., 2017).

### 2.6. Morris Sensitivity Analysis

Morris sensitivity analysis was used to understand which model parameters were sensitive (Morris, 1991). The Morris method is a global method that computes an elementary effect value for each parameter by sampling a model performance function, in this case the area under the curve for each model species in their respective timeplots, over a range of values for each parameter; the mean of elementary effects measures the direct effect of a particular parameter on the performance function, while the variance of each elementary effect indicates whether the effects are non-linear or the result of interactions with other parameters (large variance suggests connectedness or non-linearity). The Morris sensitivity coefficients were computed using the DiffEqSensitivity.jl package (Rackauckas and Nie, 2017). The parameter ranges were established by calculating the minimum and the maximum value for each parameter in the parameter ensemble generated by JuPOETs. Each range was then subdivided into 10,000 samples for the sensitivity calculation. Elementary effect values were then calculated one at a time by measuring the perturbation in the AUC on changing one parameter, where the AUC was calculated by solving the set of ODEs for each change. In order to calculate the mean and variance, the top 1000 perturbations with the highest spread in parameter values were used. In total, the model was evaluated 10000*n* times, where *n* is the number of parameters varied. The Morris sensitivity coefficients are calculated using the compute_sensitivity_coefficients.jl script in the model repositories.

## 3. Results

### 3.1. Modeling and Analysis of the C1 Circuit

**Figure 2:**
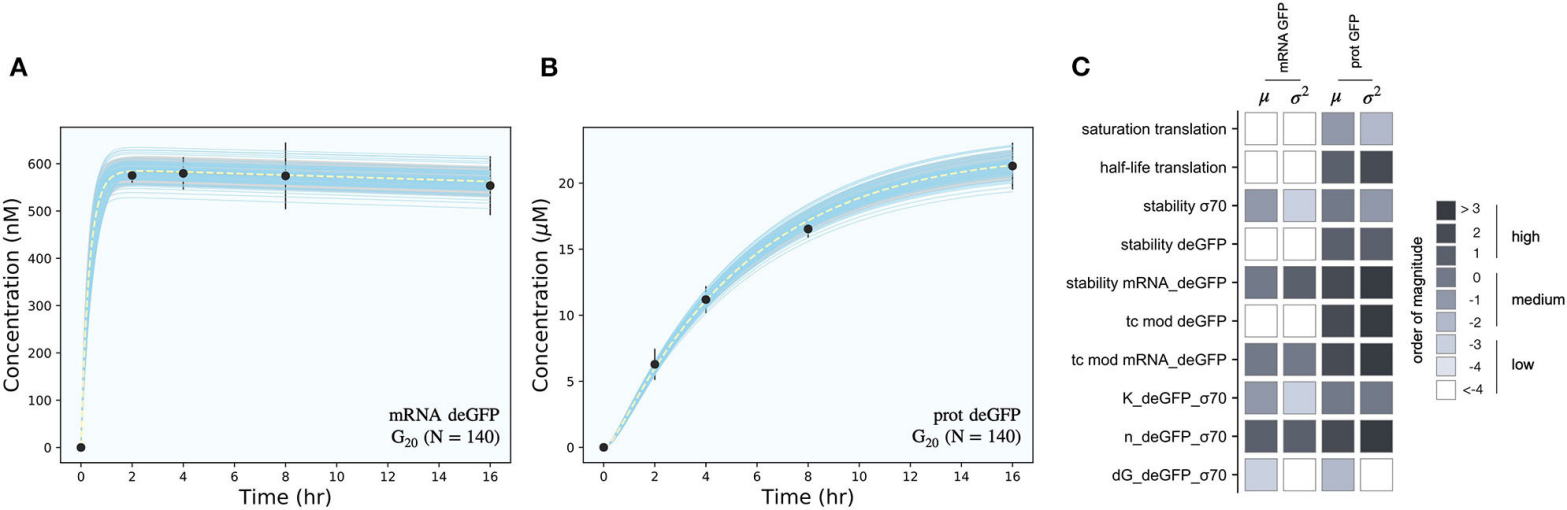
Model simulations vs. experimental measurements for σ_70_ induced deGFP expression. **(A)**: Simulated and measured deGFP mRNA concentration vs. time using the small circuit *G*_20_ ensemble (*N* = 140). **(B)**: Simulated and measured deGFP protein concentration vs. time using the small circuit *G*_20_ ensemble (*N* = 140). **(C)**: Global sensitivity analysis of the P70-deGFP circuit parameters. Morris sensitivity coefficients were calculated for the unknown model parameters, where the range for each parameter was established from the ensemble. Uncertainty: Simulations and uncertainty quantification are shown for the generation 20 (*G*_20_) ensemble which yielded *N* = 140 parameter sets that were rank two or below. The parameter ensemble was used to calculate the mean (dashed line) and the 95% confidence estimate of the simulation (gray region). Additionally, the 99% confidence estimate of the mean simulation is shown in orange. Individual parameter set trajectories are shown in blue. Points denote the mean experimental measurement while error bars denote the 95% confidence estimate of the experimental mean computed from at least three replicates.

The effective biophysical transcription and translation model captured σ_70_ induced deGFP expression at the mRNA and protein level within the experimental error for C1 (Figure 2). JuPOETs produced an ensemble (*N* = 140) of the 11 unknown model parameters which captured the transcription of mRNA (Figure 2A) and the translation of deGFP protein (Figure 2B). The mean and standard deviation of key parameters is given in Table 2. The deGFP mRNA reached its steady state concentration of approximately 580 nM within 2 h, and stayed at this level for the remainder of the reaction. Thus, the cell free reaction maintained continuous transcriptional activity with an average mRNA lifetime of 27 min; Garamella et al. (2016) reported a similar lifetime of 17–18 min. On the other hand, deGFP protein concentration increased more slowly, and began to saturate between 8 and 10 h at approximately 15 μM. Given there was negligible protein degradation (the mean deGFP half-life was estimated to be ~11 days, which was similar to the value of 6 days estimated by Horvath et al., albeit in a different cell free system, Horvath et al., 2020). The saturating protein concentration suggested that the translational capacity of the cell free system decreased over the course of the reaction. The decrease in translational capacity, which could stem from several sources, was captured in the simulations using a monotonically decreasing translation capacity state variable ϵ, and the translational control variable *w*(…). In particular, the mean half-life of translational capacity was estimated to be τ_*L*,1/2_ ~ 4 h in the C1 experiments. Taken together, JuPOETs produced an ensemble of model parameters that captured the experimental training data. Next, we considered which C1 model parameters were important to the model performance using Morris sensitivity analysis, a global sensitivity analysis method.

**Table 2:** Estimated parameter values for the P70-deGFP model (C1).

**Description**	**Parameter**	**Value (μ ± σ)**	**Units**
Translation saturation coefficient	*K*_*L*_	483.13 ± 10.10	μM
Half-life translation	τ_*L*,1/2_	4.03 ± 0.031	h^-1^
**Time constants**			
deGFP transcription	τ_X,GFP_	0.61 ± 0.04	dimensionless
deGFP translation	τ_L,GFP_	0.16 ± 0.003	dimensionless
**mRNA and protein half-life**			
mRNA deGFP	ln(2)/θ_*m, GFP*_	13.5 ± 2.47	min
Protein deGFP	ln(2)/θ_*p, GFP*_	10.86 ± 0.78	days
Protein σ_70_	ln(2)/θ_*p*,σ_70__	3.65 ± 0.17	days
**Free energies**			
RNAP + deGFP gene	Δ*G*_GFP, RX_	28.82 ± 1.75	kJ mol^-1^
RNAP + σ_70_ + deGFP gene	Δ*G*_GFP,σ_70__	–20.38 ± 1.91	kJ mol^-1^
**Binding parameters**			
Hill coefficient deGFP gene + σ_70_	*n*_*GFP*,σ_70__	1.12 ± 0.06	dimensionless
Dissociation constant deGFP gene + σ_70_	*K*_*GFP*,σ_70__	24.19 ± 2.18	μ*M*

*The mean and standard deviation of each parameter value was calculated over the ensemble of parameter sets meeting the rank selection criteria (N = 139)*.

The importance of C1 model parameters was quantified using Morris sensitivity analysis (Figure 2B). The Morris method computes the influence of each parameter, known as the elementary effect, on a model performance function. The mean of elementary effects measures the direct effect of a particular parameter, while the variance indicates whether the effects are non-linear or the result of interactions with other parameters (large variance suggests non-linearity). The performance function was defined as the integrated area under the curve (AUC) for each mRNA and protein species in their respective timeplots, calculated for each parameter value range. The Morris sensitivity measures (mean and variance) were binned into categories based upon their relative magnitudes, from no influence (white) to high influence (black). Only four parameters (translation saturation coefficient *K*_*L*_, translational capacity half-life τ_*L*,1/2_, translation time constant, and protein degradation constant) influenced the protein level. On the other hand, six parameters influenced both mRNA and protein abundance; all six of these parameters were either directly or indirectly associated with transcription. Thus, these parameters influenced the production or stability of mRNA which in turn influenced the protein level. In particular, the mRNA degradation constant and the cooperativity of σ_70_ in the P70a promoter function had the largest direct effect and variance. Surprisingly, the Δ*G* of σ_70_/RNAP/promoter configuration was the least influential of the six parameters and had a small elementary effect variance. Taken together, Morris sensitivity analysis of the C1 model parameters highlighted the hierarchical structure of the transcriptional and translational model, suggesting experimentally tunable parameters such as mRNA stability were globally important. Next, we used the ensemble of P70a, time constant and degradation parameters estimated for C1 to constrain the analysis of C2.

### 3.2. Modeling and Analysis of the C2 Circuit

**Figure 3:**
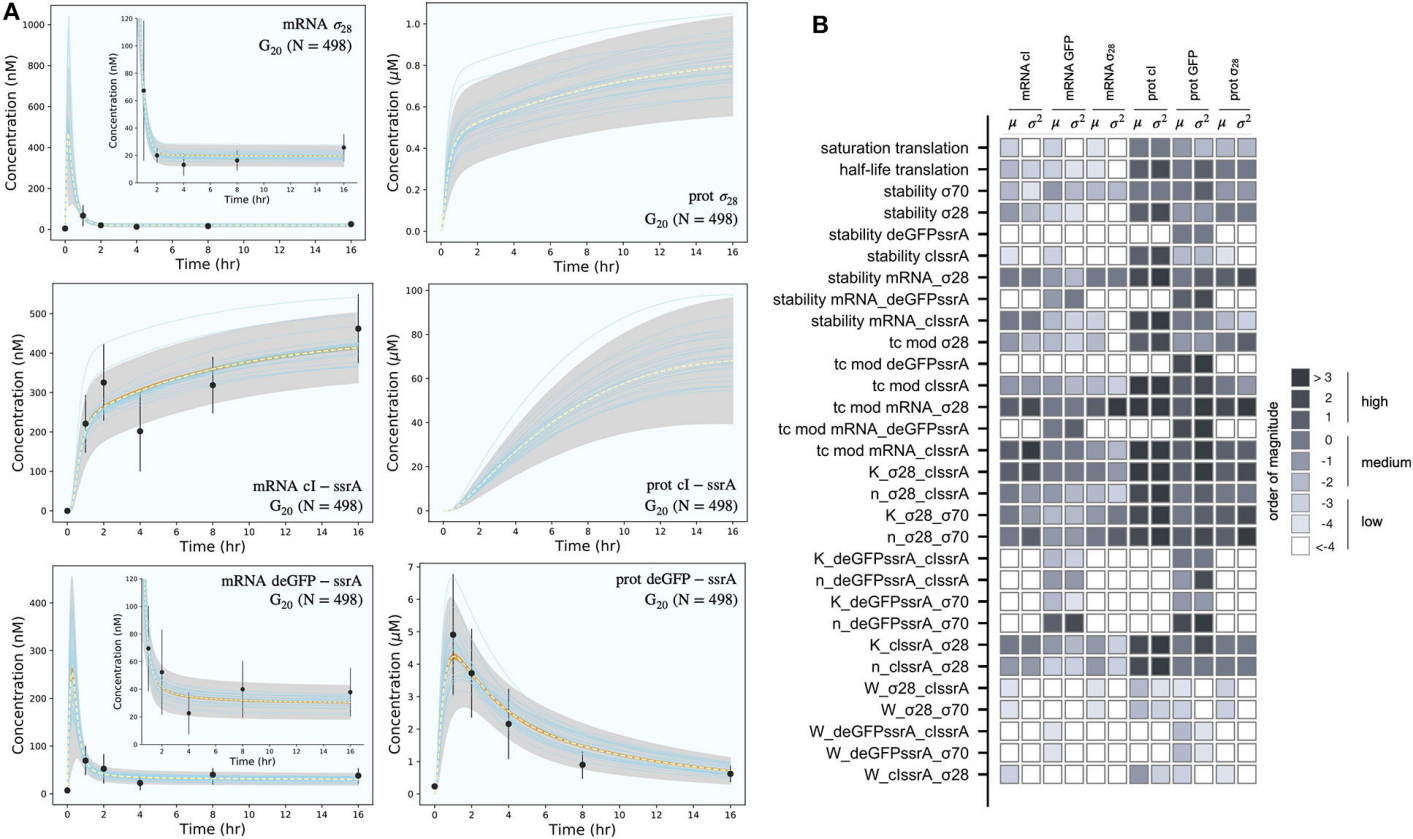
Model simulations vs. experimental measurements for the negative feedback deGFP-ssrA circuit. **(A)**: Model simulations of the negative feedback deGFP-ssrA circuit using the *G*_20_ ensemble (*N* = 498). Uncertainty: Simulations and uncertainty quantification are shown for the generation 20 (*G*_20_) ensemble which yielded *N* = 489 parameter sets (rank two or below). The parameter ensemble was used to calculate the mean (dashed line) and the 99% confidence estimate of the simulation (gray region). Additionally, the 99% confidence estimate of the mean simulation is shown in orange. Individual parameter set trajectories are also shown in blue. Points denote the mean experimental measurement while error bars denote the 95% confidence estimate of the experimental mean computed from at least three replicates. **(B)**: Global sensitivity analysis of the negative feedback deGFP-ssrA circuit parameters. Morris sensitivity coefficients were calculated for the unknown model parameters, where the range for each parameter was established from the ensemble.

The effective biophysical transcription and translation model captured the deGFP-ssrA expression dynamics in the negative feedback circuit C2 (Figure 3A). JuPOETs produced an ensemble (*N* = 498) of the 33 unknown model parameters which captured transcription and translation dynamics for σ_28_, cI-ssrA and deGFP-ssrA. The mean and standard deviation of key parameters is given in Table 3. Compared with the estimated parameters for C1, the C2 model had almost a two fold change in the half life of translation and the translation saturation coefficient. Similarly, there were variations in the values of the transcription and translation time constants for the two systems. However, for both circuits, the small values of the transcription and translation time constants qualitatively suggested elongation limited reactions; the exception was σ_28_ translation which was closer to initiation limited. Unlike C1, the mRNA expression pattern for σ_28_ and deGFP-ssrA both showed an initial spike, to a concentration similar with the previous pseudo steady state, before the cI-ssrA regulator protein could be expressed. However, once cI-ssrA began to accumulate, the concentrations of the regulated mRNAs dropped by approximately an order of magnitude compared with the unregulated case. Again, as shown with C1, the regulated mRNA concentrations reached an approximate steady-state. This further confirmed continuous transcription and mRNA degradation throughout the cell free reaction. The mean estimated mRNA lifetime for cI-ssrA and deGFP were similar (approximately 16 min), while the degradation of σ_28_ mRNA was predicted to be slower (mean mRNA lifetime was estimated to be approximately 30 min). Lastly, the mean peak degradation rate for GFP was approximately 47 nM/min, while the mean peak cI-ssrA degradation rate was predicted to be approximately 63 nM/min; both of these degradation rate estimates were consistent with the previously reported range of 15–150 nM/min measured by Garamella et al. (2016).

**Table 3:** Estimated parameter values for the negative feedback circuit (C2).

**Description**	**Parameter**	**Value (μ ± σ)**	**Units**
Translation saturation coefficient	*K*_*L*_	253.75 ± 14.12	μM
Half-life translation	τ_*L*,1/2_	8.86 ± 0.85	h^-1^
**Time constants**			
cI-ssrA transcription	τ_X,cI_	<0.001	dimensionless
deGFP transcription	τ_X,GFP_	0.045 ± 0.003	dimensionless
σ_28_ transcription	τ_X,σ_28__	0.0018 ± 0.0003	dimensionless
cI-ssrA translation	τ_L,cI_	0.054 ± 0.004	dimensionless
deGFP translation	τ_L,GFP_	0.058 ± 0.007	dimensionless
σ_28_ translation	τ_L,σ_28__	1.1 ± 0.13	dimensionless
**mRNA and protein half-life**			
mRNA cI-ssrA	ln(2)/θ_*m,cI*_	8.1 ± 0.60	min
mRNA deGFP	ln(2)/θ_*m,GFP*_	7.74 ± 1.13	min
mRNA σ_28_	ln(2)/θ_*m*,σ_28__	14.96 ± 1.60	min
Protein cI-ssrA	ln(2)/θ_*p,cI*_	0.46 ± 0.043	days
Protein deGFP-ssrA	ln(2)/θ_*p,GFP*_	0.051 ± 0.002	days
Protein σ_28_	ln(2)/θ_*p*,σ_28__	7.65 ± 0.91	days
Protein σ_70_	ln(2)/θ_*p*,σ_70__	14.86 ± 2.30	days
**Free energies**			
RNAP + cI gene	Δ*G*_cI,RX_	46.57 ± 4.28	kJ mol^-1^
RNAP + σ_28_ + cI gene	Δ*G*_cI,σ_28__	−1.10 ± 0.04	J mol^-1^
RNAP + deGFP gene	Δ*G*_GFP,RX_	41.94 ± 1.80	kJ mol^-1^
RNAP + σ_70_ + deGFP gene	Δ*G*_GFP,σ_70__	−27.67 ± 1.79	kJ mol^-1^
RNAP + cI + deGFP gene	Δ*G*_GFP,cI_	−7.21 ± 1.14	kJ mol^-1^
RNAP + σ_28_ gene	Δ*G*_σ_28_,RX_	46.67 ± 3.18	kJ mol^-1^
RNAP + σ_70_ + σ_28_ gene	Δ*G*_σ_28_,σ_70__	−10.46 ± 1.15	kJ mol^-1^
RNAP + cI + σ_28_ gene	Δ*G*_σ_28_,cI_	−12.89 ± 1.44	kJ mol^-1^
**Hill coefficients**			
cI gene + σ_28_	*n*_*cI*,σ_28__	1.88 ± 0.28	dimensionless
deGFP gene + σ_70_	*n*_*GFP*,σ_70__	1.53 ± 0.14	dimensionless
deGFP gene + cI	*n*_*GFP,cI*_	0.698 ± 0.133	dimensionless
σ_28_ gene + σ_70_	*n*_σ_28_,σ_70__	1.10 ± 0.10	dimensionless
σ_28_ gene + cI	*n*_σ_28_,*cI*_	1.51 ± 0.25	dimensionless
**Dissociation constants**			
cI gene + σ_28_	*K*_*cI*,σ_28__	1.09 ± 0.088	μ*M*
deGFP gene + σ_70_	*K*_*GFP*,σ_70__	86.87 ± 7.13	μ*M*
deGFP gene + cI	*K*_*GFP,cI*_	3.83 ± 0.41	μ*M*
σ_28_ gene + σ_70_	*K*_σ_28_,σ_70__	1.35 ± 0.26	μ*M*
σ_28_ gene + cI	*K*_σ_28_,*cI*_	0.0389 ± 0.0068	μ*M*

*The mean and standard deviation for each parameter was calculated over the ensemble of parameter sets (N = 498)*.

The secondary effect of cI-ssrA repression was visible in the cI-ssrA mRNA expression pattern. The expression of cI-ssrA was induced by σ_28_, however, σ_28_ expression was repressed by cI-ssrA, thereby completing a negative feedback loop. Initially, before appreciable levels of cI-ssrA had been translated, the cI-ssrA transcription rate was maximum (approximately 200 nM/h). However, the transcription rate decreased to approximately 12 nM/h after 2 h and remained constant for the remainder of the cell free reaction. Similarly, transcription rates for σ_28_ (approximately 1,200 nM/h) and deGFP-ssrA (approximately 750 nM/h) were initially at a maximum due to the presence of endogenous σ_70_, but then quickly dropped as cI protein accumulated. Protein synthesis followed a similar trend, with the translation rates for σ_28_ and deGFP-ssrA initially present at their maximum values before quickly dropping. After 1 h, deGFP levels reached a peak and decayed due to the ClpXP-mediated degradation, whereas σ_28_ protein levels continued to slowly rise at a steady rate (approximately 15 nM/h). The C2 model also predicted the expected lag present during the initial phase of cI-ssrA protein synthesis due to the need for σ_28_ protein to reach appreciable levels. Moreover, the combination of high cI-ssrA mRNA abundance (expressed because σ_28_ does not have a degradation tag) and ClpXP-mediated degradation led to the saturation of the cI-ssrA protein concentration. However, the cI-ssrA protein concentration could not be verified because we did not have an experimental measurement for this species. Taken together, the effective model simulated cell free expression dynamics for C2. Next, we considered which C2 model parameters were important using Morris sensitivity analysis.

Morris sensitivity analysis of the negative feedback circuit C2 stratified the parameters into locally and globally important groups (Figure 3B). The influence of 33 parameters was computed using the AUC of each mRNA and protein species as the performance function. The Morris sensitivity metrics (mean and variance) were binned into categories based upon their relative magnitudes, from no influence (white) to high influence (black). Some parameters affected only their respective mRNA or protein target, whereas others had widespread effects. For example, the time constant (tc) modifiers, stability of deGFP-ssrA protein and mRNA, and the binding dissociation constant (K) and cooperativity parameter (n) of cI-ssrA and σ_70_ for the deGFP-ssrA promoter affected only the values of deGFP-ssrA protein and mRNA. On the other hand, the tc, stability, K and n parameters for σ_70_, σ_28_, or cI-ssrA influenced mRNA and protein expression globally. The σ_70_ and σ_28_ proteins acted as inducers or repressors for more than one gene product: σ_70_ induced both deGFP-ssrA and σ_28_, and cI-ssrA protein repressed both of these genes. Degradation constants (denoted as stability) affected the half-lives of the transcribed messages or the translated proteins in the mixture, while the time constant modifiers changed the time required to form the open gene complex (or translationally active complex). Dissociation and cooperativity constants affected the binding interactions of the inducer (or repressor in the case of cI-ssrA) in the promoter control function. Varying these parameters, therefore, had a strong effect on their respective targets. Similarly, the translation saturation and its half-life, which captured the depletion in the translation activity over the course of the reaction, not only affected protein levels but also mRNA levels. This is because these parameters tuned the rate of formation of cI-ssrA, which in turn affected the mRNA levels of its gene targets. Given that cI-ssrA was the main regulator (repressor) of the circuit, the parameters that dictated the levels of cI-ssrA mRNA and protein had a global effect. We also observed high sensitivity variance for several parameters, in particular involving cI-ssrA. For example, the time constant modifiers for cI-ssrA mRNA and protein had a two-pronged effect. On the one hand, they positively influenced the transcription/translation rates of the gene and mRNA products, directly increasing the cI-ssrA protein. On the other hand, increased cI-ssrA expression reduced the level of σ_28_, in turn reducing the cI-ssrA levels over time. Taken together, Morris sensitivity analysis of the C2 model stratified that parameters into local and globally important groups, with the parameters governing the synthesis rates of the cI-ssrA mRNA and protein being globally important. The sensitivity analysis also gave insight into the organization of the circuit, suggesting cI to be highly connected within the circuit.

## 4. Discussion

In this study, we developed an effective biophysical modeling approach to simulate transcription (TX) and translation (TL) processes occurring in a cell free system. We tested this approach by simulating the dynamics of two cell free synthetic circuits (C1 and C2).

The model formulation, and parameter values were mechanistic and largely derived from literature. For example, characteristic values for τ_*X*_ and *K*_*X*_, the time and saturation constants for transcription, were approximated from *in vitro* experiments using an abortive initiation assay (McClure, 1980). The RNAP and ribosome elongation rates were taken from Garamella et al. (2016), while other parameters were estimated from BioNumbers (Milo et al., 2010). Similarly, the weights appearing in the transcription control function *u*(…) were based upon the Gibbs energies of the respective promoter configurations, while the form of the transcriptional control functions was derived from a statistical mechanical treatment of promoter activity (Ackers et al., 1982; Lee and Bailey, 1984; Moon et al., 2012). However, there were parameters that were not available from literature; in these cases multiobjective optimization was used to estimate these parameters from mRNA and protein measurements. For C1, sigma factor 70 (σ_70_) induced expression of green fluorescent protein (deGFP), the time constants, degradation rates, and other parameters governing deGFP expression were estimated from measurements of deGFP mRNA and protein. These estimates were then used to constrain the parameter search for C2, which involved deGFP expression subject to negative feedback and programmed protein degradation. We estimated which model parameters were important to the performance of C1 and C2 using Morris sensitivity analysis. Sensitivity analysis results for C1 were expected; the time constant for transcription, the stability of the deGFP message and the cooperativity of σ_70_ were all important parameters. On the other hand, the sensitivity analysis results for C2 were more nuanced, with parameters (and associated species) being stratified into locally and globally important groups; the performance of C2 was most sensitive to the parameters controlling the cI-ssrA mRNA and protein abundance.

The effective TX/TL modeling approach described here has several potential applications. For example, a challenge of *in vivo* constraint based modeling is the description of gene expression (Covert and Palsson, 2002). Boolean and probabilistic approaches (Covert et al., 2001, 2004; Chandrasekaran and Price, 2010) have been developed to address this challenge. However, the transcriptional state of a boolean gene is either on or off based on the state of its regulators, thus, gene expression is coarse-grained. The current modeling approach could be an interesting mechanistic alternative to the boolean framework that utilizes a continuous description of gene expression dynamics and transcriptional regulation. In particular, the rules encoding typical boolean gene expression networks are easily translatable into the rational promoter functions described here, however, the estimation of the parameters appearing in these promoter functions, especially in an *in vivo* context, remains an open question. Another application could be the extension of the current model to other prokaryotic or eukaryotic systems with a few changes. For example, in order to adopt it for an *in vivo* system, the dilution of resources due to growth (proportional to the cellular doubling time) would be added as a first order term to the mRNA and protein balance equations. Additionally, the competition for RNAP and ribosomes, denoted respectively as $O_{X,j}$ and $O_{L,j}$ in the study, and assumed to be negligible due to the presence of only three genes in the system, would need to be taken into account; this term would serve to change the rates of transcription and translation of the added genes because of the presence of a large amount of endogenous genes in the *in vivo* system. Moreover, characteristic literature-based parameter values would be different for cellular processes compared to the *in vitro* ones used in this study, and they would thus need to be adjusted accordingly. For the case of a mammalian or a yeast *in vivo* system, a few more changes to the current model are necessary because the mechanistic processes of gene expression and regulation are different in these two types of systems. For example, a key difference present in eukaryotes is the addition of an intron splicing step during the synthesis of a mature mRNA from a pre-mRNA. In addition, the gene regulation mechanisms are vast and composed of numerous elements in eukaryotes. Finally, especially in *in vivo* systems, addition of exogenous genes often leads to a tug-of-war of carbon and energy resources between cellular growth processes and the expression of these genes, driving cellular resources away from the latter. Synthetic biology studies often neglect the role that metabolism plays in the expression of synthetic circuits. Ultimately, metabolism is centrally important to the operation of any synthetic circuit; gene expression is strongly dependent upon the metabolic resources provided by catabolic processes. It is imperative that this metabolic burden by the addition of exogenous genes be incorporated in the *in vivo* model description to accurately capture the expression behavior. We have recently started to explore this question by integrating effective transcription and translation models with metabolic networks in cell free reactions e.g., Vilkhovoy et al., 2018; Horvath et al., 2020, and also developing experimental tools to measure metabolite concentrations in cell free systems (Vilkhovoy et al., 2019). However, these previous transcriptional and translational models (and similar precursor models simulating eukaryotic processes, Gould et al., 2016; Tasseff et al., 2017) were less developed than those presented here. Taken together, the effective modeling approach described here can potentially be used to simulate transcription and translation processes in a variety of applications.

There have been many studies looking into oscillatory and other dynamic behavior of synthetic circuits (see Prangemeier et al., 2020). A negative feedback loop, such as the one explored here, has the potential to give rise to oscillations. Yelleswarapu et al. carried out TX/TL reactions, with a circuit similar to C2, in both batch and continuous conditions (Yelleswarapu et al., 2018). Similar to our study, no oscillations were observed in the batch reaction. However, oscillations were observed in the continuous reaction. There are several possible reasons why no oscillations were seen in our (or the Yelleswarapu et al.) batch study; as it was carried out in batch, dilution of the expressed protein or mRNA species due to an inlet feed was not possible. Thus, mRNA species reached a pseudo steady state (after approximately 2 h) because of ribonuclease degradation (Garenne et al., 2019). On the other hand, in general protein species were not at steady-state; only proteins tagged with a ssrA tag were able to be degraded by the ClpXP system, thereby allowing a steady-state. Thus, the batch system likely evolved dynamically through a set of concentration profiles that were not consistent with oscillations.

The effective TX/TL model described the experimental mRNA and protein training data. However, there were several important questions to be addressed by future studies. First, the model formulation described the data, but did not predict dynamics outside of the training set. If this approach is to be useful to the synthetic biology community, or more broadly as an effective biophysical technique to model *in vivo* gene expression dynamics for applications such as regulatory flux balance analysis, we need to have confidence that the modeling approach is predictive. Thus, while we have established a descriptive model, we have yet to establish a predictive model. Next, there were several technical or mechanistic questions that should be explored further. For example, cI-ssrA represses the activity of the P70a promoter via interaction with its OR2 and OR1 operator sites; in this study we considered only a single operator site suggesting that we potentially underestimated the potency of cI repression in the deGFP and σ_28_ promoter functions, see the multiplication rule (Lucks et al., 2011). Further, we used a first order approximation of ClpXP mediated protein degradation, while Garamella et al. (2016) described this degradation as zero order. Similarly, we did not establish the concentration of ClpXP in the commercially available cell free reaction mixture. The levels of this protein complex could be an important factor controlling protein degradation. Next, we should compare the current modeling approach, and the values estimated for the model parameters, with the study of Marshall and Noireaux (2019). For example, one of the potential limitations of the current study (that was addressed by Marshall and Noireaux, 2019) is that we did not consider a separate species for dark GFP. In our previous RNA circuit modeling (Hu et al., 2015), we did include this term, but failed to do so here. We expect inclusion of a dark vs. light GFP species could influence the values for the estimated parameters, particularly the translation time constants. However, previous reports suggested the *in vitro* maturation time of deGFP was approximately 8 min (Shin and Noireaux, 2010), much faster than the typical maturation times for GFP of 1 h *in vivo* (Sniegowski et al., 2005; Iizuka et al., 2011). Thus, the impact of including a dark vs. light GFP species may not be worth the increased model complexity. Lastly, we should validate the values estimated for the binding function parameters and the promoter configuration free energies. Maeda et al. measured the binding affinities of the seven *E. coli* σ factors with RNAP (Maeda et al., 2000); while not directly comparable, these measurements give an order of magnitude characteristic value for the dissociation constants appearing in the promoter binding functions. Further, there have been several studies that have quantified the binding energies of promoter configurations (e.g., Ackers et al., 1982; Brewster et al., 2012; Tapia-Rojo et al., 2012, 2014). A perfunctory inspection of the values estimated in this study suggested our estimated free energy values, while the same order of magnitude as previous studies in many cases, did have values that were off by a factor of up to an order of magnitude compared with literature (albeit for different promoters). In particular, the positive Gibbs energy estimated for free RNAP binding leading to transcription was likely too large, while the magnitude of other values such as the energy of cI repression of σ_28_ expression was likely too small. Thus, these other studies could serve as a basis to validate our estimates, and perhaps more importantly constrain the parameter search space for future studies.

## Data Availability Statement

Model code is available under an MIT software license from the Varnerlab GitHub repository for TXTL model code (https://github.com/varnerlab/Biophysical-TXTL-Model-Code). The mRNA and protein measurements presented in this study are available in the data directory of the model repositories in comma separated value (CSV) and Microsoft Excel format.

## Author Contributions

JV directed the study. MV, SV, HL, and AA conducted the cell free experimental measurements. JV, MV, and AA developed the reduced order models and the parameter ensemble. MV, AA, and JV analyzed the model ensemble, and generated figures for the manuscript. The manuscript was prepared and edited for publication by AA, MV, SV, and JV. All authors reviewed this manuscript.

## Conflict of Interest

The authors declare that the research was conducted in the absence of any commercial or financial relationships that could be construed as a potential conflict of interest.

## Footnotes

^1^Varnerlab. Github reposistory for tx/tl model code. Available online at https://github.com/varnerlab/Biophysical-TXTL-Model-Code.

^2^Varnerlab. Gene Regulatory Network Generator in Julia (JuGRN). Available online at https://github.com/varnerlab/JuGRN-Generator.
