# Supplementary material for: Effective Biophysical Modeling of Cell Free Transcription and Translation Processes
Source: Front Bioeng Biotechnol. 2020 Nov 26;8:539081. doi: 10.3389/fbioe.2020.539081 (PMC7726328; doi:10.3389/fbioe.2020.539081)
Supplement: Supplementary file 1 [file Data_Sheet_1.pdf]

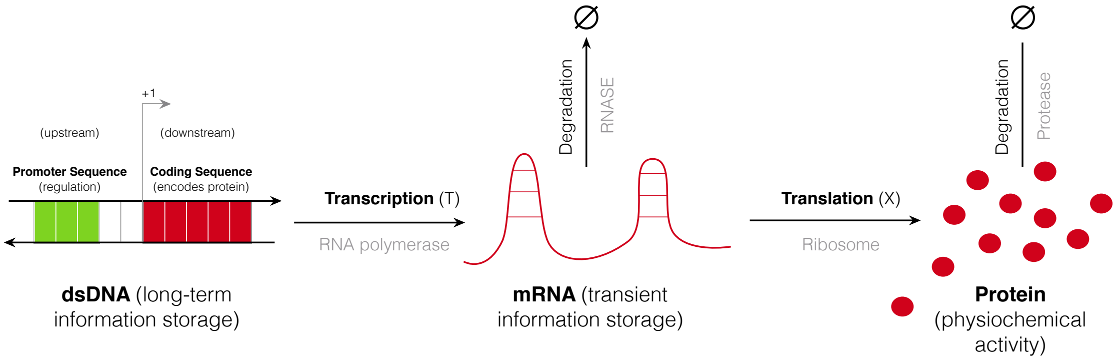

Fig. S1: Schematic of transcriptional (TX) and translation (TL) processes.

Effective expressions for transcription (and translation) kinetics

To develop expressions for $r_{X,i}$ (or $r_{L,i}$) let us develop a mental model of the elementary steps occurring in transcription (Fig. S1). Our mental model for transcription, based upon the earlier work by McClure [48] and later Bailey [44], consists of a four step elementary reaction scheme:

$$\mathcal{G}_j + R_X \rightleftharpoons (\mathcal{G}_j : \mathrm{R_X})_C \quad \text{(S1)}$$

$$(\mathcal{G}_j : \mathrm{R_X})_C \longrightarrow (\mathcal{G}_j : \mathrm{R_X})_O \quad \text{(S2)}$$

$$(\mathcal{G}_j : \mathrm{R_X})_O \longrightarrow R_X + \mathcal{G}_j \quad \text{(S3)}$$

$$(\mathcal{G}_j : \mathrm{R_X})_O \longrightarrow m_j + R_X + \mathcal{G}_j \quad \text{(S4)}$$

where $\mathcal{G}_j$, R_X denote the gene and *free* RNA polymerase (RNAP) concentration, and $(\mathcal{G}_j : \mathrm{R_X})_O$, $(\mathcal{G}_j : \mathrm{R_X})_C$ denote the open and closed complex concentrations, respectively. Let the kinetic rate of transcription be directly proportional to the concentration of the open complex:

$$r_{X,j} = k_{E,j}^{X}\,(\mathcal{G}_j : \mathrm{R_X})_O$$

where $k_{E,j}^{X}$ is the elongation rate constant for gene j. The key idea behind this derivation is that the RNAP (or Ribosome) acts as an enzyme. Thus, we might expect that we could use a strategy similar to enzyme kinetics to derive an expression for $r_{X,j}$ (and $r_{L,j}$). The material balances around the closed and open complex for gene j are given by:

$$\begin{aligned}\frac{d}{dt}\left(\mathcal{G}_j:\mathrm{R_X}\right)_C &= k_{+}\left(\mathcal{G}_j\right)\left(\mathrm{R_X}\right)-k_{-}\left(\mathcal{G}_j:\mathrm{R_X}\right)_C-k_{I}\left(\mathcal{G}_j:\mathrm{R_X}\right)_C && \text{(S5)}\\ \frac{d}{dt}\left(\mathcal{G}_j:\mathrm{R_X}\right)_O &= k_{I}\left(\mathcal{G}_j:\mathrm{R_X}\right)_C-k_{A}\left(\mathcal{G}_j:\mathrm{R_X}\right)_O-k_{E,j}^{X}\left(\mathcal{G}_j:\mathrm{R_X}\right)_O && \text{(S6)}\end{aligned}$$

where k_{+} (conc^{-1} t^{-1}) and k_{-} (t^{-1}) denote the on/off rate constant for RNAP at the promoter for gene j, k_I (t^{-1}) denotes the rate constant governing open complex formation and k_A (t^{-1}) denotes the rate constant governing abortive initiation. The total abundance of RNAP, denoted as $R_{X,T}$ is governed by:

$$R_{X,T}=R_X+\left(\mathcal{G}_j:\mathrm{R_X}\right)_C+\left(\mathcal{G}_j:\mathrm{R_X}\right)_O \tag{S7}$$

At steady state, the abundance of the closed and open complexes can be estimated from the balance equations (where we have neglected the subscript j for simplicity):

$$\begin{aligned}\left(\mathcal{G}_j:\mathrm{R_X}\right)_C &\simeq \left(\frac{k_{+}}{k_{-}+k_{I}}\right)\left(\mathcal{G}_j\right)\left(\mathrm{R_X}\right) && \text{(S8)}\\ \left(\mathcal{G}_j:\mathrm{R_X}\right)_O &\simeq \left(\frac{k_{I}}{k_{A}+k_{E}^{X}}\right)\left(\mathcal{G}_j:\mathrm{R_X}\right)_C && \text{(S9)}\end{aligned}$$

The ratio of parameters in the open and closed complex expressions have special significance which is apparent from looking at their units. For example, the ratio:

$$K_{X,j}^{-1}\equiv\left(\frac{k_{+}}{k_{-}+k_{I}}\right) \tag{S10}$$

is a saturation constant for gene j with units of concentration, while:

$$\tau_{X,j}^{-1} \equiv \left(\frac{k_I}{k_A + k_E^X}\right) \tag{S11}$$

is a time constant for gene j comparing the initiation, abortive initiation and elongation constants. We can relate the open complex to the concentration of gene j and *free* RNAP concentration by eliminating the closed complex concentration from the steady state expressions:

$$\left(\mathcal{G}_j : \mathrm{R_X}\right)_O \simeq (K_{X,j}^{-1})(\tau_{X,j}^{-1})\left(\mathcal{G}_j\right)\left(\mathrm{R_X}\right) \tag{S12}$$

To estimate the *free* RNAP concentration we can use the total RNAP balance, where we have substituted expressions for the open and closed complex concentrations:

$$R_{X,T} = R_X + (K_{X,j}^{-1})\left(\mathcal{G}_j\right)\left(\mathrm{R_X}\right) + (K_{X,j}^{-1})(\tau_{X,j}^{-1})\left(\mathcal{G}_j\right)\left(\mathrm{R_X}\right) \tag{S13}$$

Starting with Eqn (S13), solving for *free* RNAP concentration R_X gives:

$$R_X = \frac{R_{X,T}\left(\tau_{X,j}K_{X,j}\right)}{\tau_{X,j}K_{X,j} + \left(\tau_{X,j}+1\right)\mathcal{G}_j} \tag{S14}$$

Now that we have R_X we can get the open complex concentration in terms of total RNAP:

$$\left(\mathcal{G}_j : \mathrm{R_X}\right)_O \simeq \frac{R_{X,T}\mathcal{G}_j}{\tau_{X,j}K_{X,j} + \left(\tau_{X,j}+1\right)\mathcal{G}_j} \tag{S15}$$

Lastly, the kinetic rate of transcription is proportional to the open complex concentration which can now be substituted to give:

$$r_{X,j} = k_{E,j}^X R_{X,T}\left(\frac{\mathcal{G}_j}{\tau_{X,j}K_{X,j} + \left(\tau_{X,j}+1\right)\mathcal{G}_j}\right) \tag{S16}$$

In an identical procedure, we can also formulate a model of the translation rate:

$$r_{L,j} = k_{E,j}^{L} R_{L,T} \left(\frac{m_j}{\tau_{L,j} K_{L,j} + (\tau_{L,j} + 1)\, m_j} \right) \tag{S17}$$

where m_j denotes the concentration of mRNA j.

Which is limiting, elongation or initiation? Ultimately, this question depends upon the gene of interest. However, we can see some interesting properties of $r_{X,j}$ by considering limiting cases for the value of the time constant $\tau_{X,j}$. Assume the rate constant for abortive initiation k_A is small compared to both k_I and $k_{E,j}^{X}$:

$$\tau_{X,j} \simeq \frac{k_{E,j}^{X}}{k_I} \tag{S18}$$

When $\tau_{X,j} \gg 1$ (initiation limited) the kinetic transcription rate becomes:

$$r_{X,j} = \frac{k_{E,j}^{X} R_{X,T}}{\tau_{X,j}} \left(\frac{\mathcal{G}_j}{K_{X,j} + \mathcal{G}_j} \right) \tag{S19}$$

while $\tau_{X,j} \ll 1$ (elongation limited) gives:

$$r_{X,j} = k_{E,j}^{X} R_{X,T} \left(\frac{\mathcal{G}_j}{K_{X,j} \tau_{X,j} + \mathcal{G}_j} \right) \tag{S20}$$

How do we get values for k_{+}, k_{-}, k_I, $k_{E,j}^{X}$ and k_A? Generally speaking, except for $k_{E,j}^{X}$ which we can estimate from first principles, estimating the value of k_{+}, k_{-}, k_I and k_A is difficult (especially *in-vivo*). Thus, let's start with $k_{E,j}^{X}$; the elongation rate constant is proportional to the elongation rate of the polymerase e_X (units of nt s^{-1}) multiplied by the length (nt) of the coding region of gene j, or $\mathcal{L}_j$ (the length of DNA the RNAP has to read). However, typically we formulate $k_{E,j}^{X}$ in a slightly different way; first, we compute an average or characteristic elongation rate constant $\left\langle k_{E}^{X} \right\rangle$, and then correct this

characteristic value by the actual length of gene j:

$$k_{E,j}^{X} = \left\langle k_{E}^{X} \right\rangle \left(\frac{\mathcal{L}}{\mathcal{L}_j} \right) \tag{S21}$$

where:

$$\left\langle k_{E}^{X} \right\rangle = e_X \mathcal{L}^{-1} \tag{S22}$$

and $\mathcal{L}$ denotes some characteristic length, e.g., the average length of genes in *E.coli.* For the other parameters, we must estimate them from experimental data.

McClure performed a series of *in vitro* experiments to estimate k_I in transcription and produced a constraint governing permissible values for the remaining transcriptional parameters [48]. In particular, McClure used an abortive initiation assay in which the production of mRNA never completed. Instead, transcription always aborted leaving a stable open complex that could be directly measured. From these measurements, and a mental transcriptional model very similar to ours, McClure developed the expression:

$$\tau_{obs} = \frac{1}{k_I} + \frac{1}{R_{X,T}} \left(\frac{k_{-} + k_I}{k_{+} k_I} \right) \tag{S23}$$

where τ_{obs} is the time required to fully form the open complex (measured) and $R_{X,T}$ denotes the total concentration of RNAP. A value for k_I, and a relationship between the other parameters, can be obtained from the intercept and slope of a $R_{X,T}^{-1}$ versus τ_{obs} plot for a particular promoter of interest.
